# A colon cancer patient with splenic metastasis associated with a splenic abscess and thrombocytopenia: A case report

**DOI:** 10.1097/MD.0000000000040936

**Published:** 2024-12-13

**Authors:** Fang Cui, Rongfeng Zhu, Zhangjun Qian, Yunlei Zhang

**Affiliations:** a Department of Oncology, The Affiliated Yixing Hospital of Jiangsu University, Wuxi, Jiangsu Province, China; b Ultrasound Medical Imaging Department, The Affiliated Yixing Hospital of Jiangsu University, Wuxi, Jiangsu Province, China.

**Keywords:** colon cancer, low platelet count, splenic abscess

## Abstract

**Rationale:**

Splenic metastases concomitant with abscesses are rare and challenging for clinicians. The primary treatment options include splenectomy and ultrasound-guided percutaneous perforation and drainage.

**Patient concerns:**

A case of splenic abscess in a colon cancer patient with splenic metastasis who developed chills and fever for approximately 2 weeks. However, the best broad-spectrum antibiotics are ineffective. Moreover, the patient had a high bleeding risk for percutaneous perforation due to a low platelet count.

**Diagnoses:**

Colon cancer, splenic metastasis, splenic abscess, thrombocytopenia.

**Interventions:**

Ultrasound-guided percutaneous splenic abscess perforation and drainage were performed after platelet transfusion and stimulation of platelet production in the bone marrow.

**Outcomes:**

His fever was immediately relieved at night and thrombocytopenia did not relapse thereafter. His platelet count increased rapidly and reached 121 × 10^9^/L 3 days later.

**Lessons:**

Spleen metastasis in tumor patients necessitates vigilance for the potential development of spleen abscess. A less invasive procedure can be feasible in cases of low platelet count without significant coagulation dysfunction. When there is no alternative therapeutic schedule, doctors should fully evaluate the risks and benefits.

## 1. Introduction

Splenic abscesses are rare, occurring in approximately 0.2% of cases.^[1]^ Cancer patients with impaired immune systems are more susceptible to developing this disease, especially during prolonged use of antitumor medications such as cytotoxic medications, which exacerbate immunosuppression.^[2]^ The therapeutic regime for treating splenic abscesses include splenectomy, ultrasound-guided percutaneous drainage, and antibiotic therapy. We present a case of a patient with colon cancer who developed splenic metastasis, subsequently experiencing a splenic abscess and a concomitant low platelet count. The patient was therefore at a significant risk of bleeding due to invasive procedures. With sufficient preoperative preparation, ultrasound-guided percutaneous splenic abscess perforation without hemorrhage was successfully performed. To the best of our knowledge, this is the first report on the treatment of a patient with a splenic abscess and low platelet count, and this study provides a reference for clinical practice.

## 2. Case presentation

**Figure 1.**
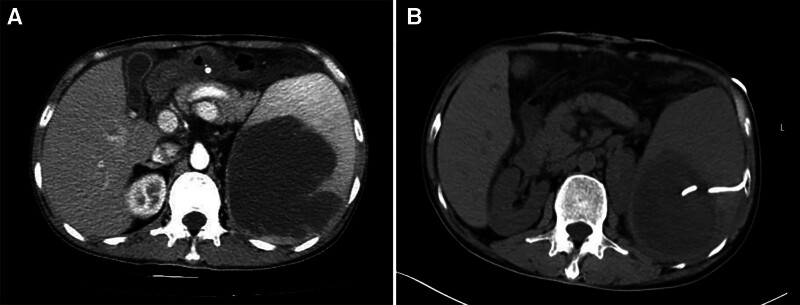
(A) Contrast-enhanced abdominal computed tomography image showing a low-density lesion in the spleen, and the lesion was considered to be a metastasis before splenic abscess formation. (B) Abdominal computed tomography image showing a drainage tube from the hole in the spleen.

**Figure 2.**
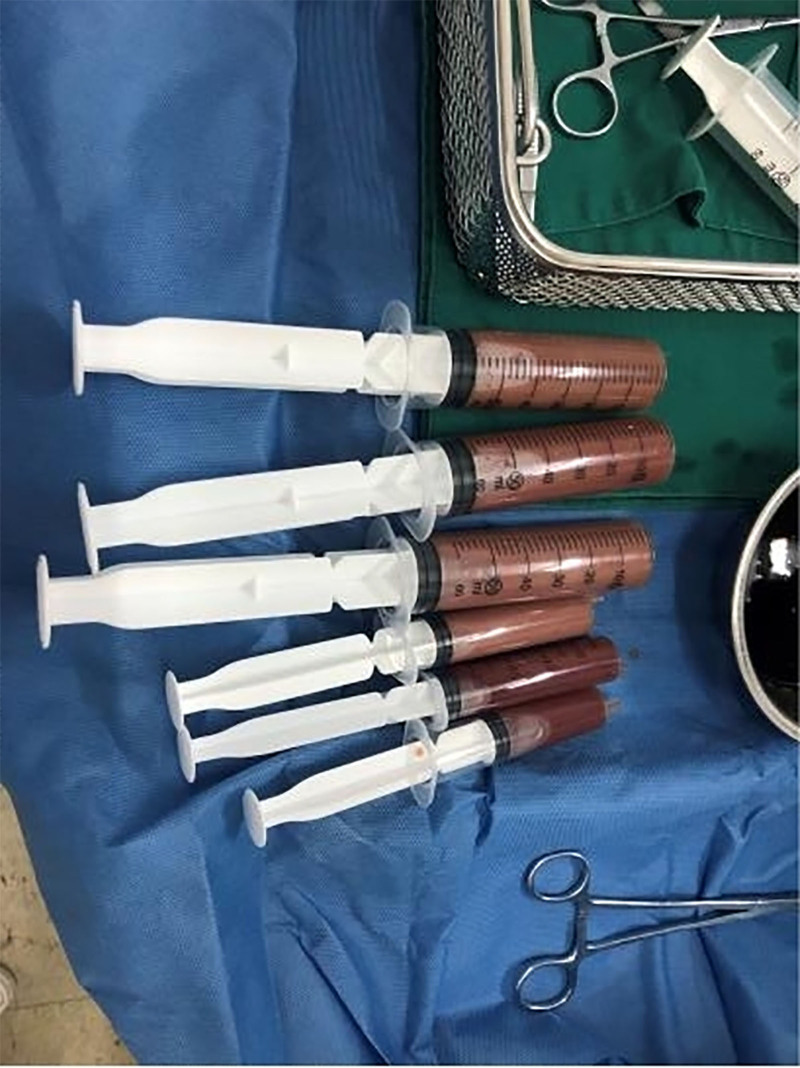
Ultrasound-guided percutaneous drainage of the splenic abscess and drainage of bloody pus.

**Figure 3.**
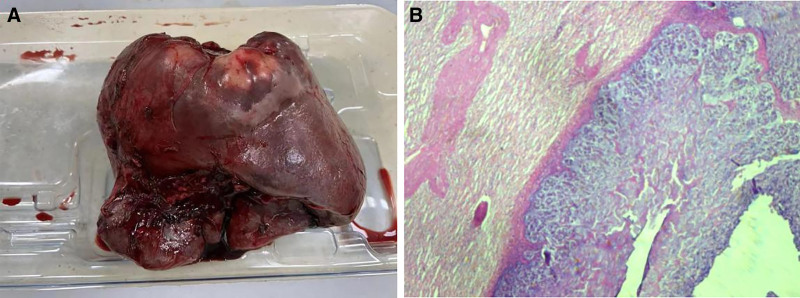
The spleen grossly after splenectomy (A) and postoperative pathology showing involvement of a mucinous adenocarcinoma (B).

Radical left colectomy was performed on a 49-year-old man on October 5, 2016. Postoperative pathology revealed a mucinous adenocarcinoma that penetrated all colonic layers. Among the 21 resected nodules, 9 had tumor cell involvement; however, there was no evidence of distant metastasis. During the 4 years, he did not undergo regular physical examinations. In February 2022, computed tomography (CT) revealed a large splenic and pelvic cystic space, with a carcinoembryonic antigen level of 369.40 ng/mL. Abdominal and pelvic metastases of left colon cancer were considered after reviewing the patient’s medical history. Genetic testing was performed for precision therapy. The results showed that the microsatellite stable type, Kirsten rat sarcoma viral oncogene homolog, neuroblastoma RAS viral oncogene homolog, and V-raf murine sarcoma viral oncogene homolog B were wild type. From February to August 2022, the patient received 12 cycles of chemotherapy (fluorouracil and oxaliplatin), along with cetuximab-targeted treatment. During this period, the disease efficacy evaluation determined that the patient had a stable disease. The patient suddenly complained of fever and abdominal discomfort on October 10, 2022, with a maximum body temperature of 40°C. A physical examination revealed a dull ache in the left middle and lower abdomen. The patient’s white blood cell count was as high as 21.3 × 10^9^/L, and his C-reactive protein level remained high at 116.13 mg/L. Combined with the CT image shown in Figure 1, we considered the diagnosis of splenic abscess. After empirical administration of broad-spectrum antibiotics for 5 days, he still experienced nighttime chills, and his body temperature increased to 41°C. Puncture and drainage were considered because of the unsatisfactory efficiency of antimicrobial therapy. Unfortunately, since the patient had chronic hepatitis B virus infection and splenomegaly, his platelet was between 70 and 100 × 10^9^/L and further decreased to as low as 12 × 10^9^/L. Fortunately, there were no significant abnormalities in coagulation function. Following platelet transfusion and stimulation of platelet production in the bone marrow, the platelet count reached 46 × 10^9^/L. After informed consent was obtained from the patient and his family, ultrasound-guided percutaneous puncture drainage was performed. The drainage fluid was bloody pus (Fig. 2). Finally, both blood and drainage pyogenic fluid cultures revealed *Bacteroides fragilis*. His fever was immediately relieved at night and thrombocytopenia did not relapse thereafter. His platelet count increased rapidly and reached 121 × 10^9^/L 3 days later. In addition, the patient’s nutritional status improved as reflected by a gradual increase in body weight. Several months later, splenectomy was performed, and postoperative pathology indicated mucinous adenocarcinoma (Fig. 3). He made a remarkable recovery. During a subsequent outpatient follow-up, the patient recounted lingering concerns about his critical condition at that time. He expressed profound gratitude for the physician’s timely and accurate decision-making. Throughout the entire treatment, the patient and his family experienced a collaborative approach to managing the illness, recognized the professionalism and dedication of the medical team, and appreciated the patient-centered care philosophy.

## 3. Discussion

Splenic abscesses are uncommon, with the majority of reported cases being case reports. One of the largest recent studies, involving 67 cases of splenic abscess in Taiwan, elucidated the clinical characteristics and prognostic factors associated with splenic abscess in this medical center. They commonly develop in immunocompromised patients. Other comorbidities were also recorded, including HIV infection, immunosuppressive therapy, neoplasia, splenic metastasis or infarction, and diabetes mellitus.^[3,4]^ Research has revealed an association between splenic abscesses and tumors. Spleen abscesses can occur from malignancies in the splenic flexure region^[5]^ and are often caused by mucinous colon tumors.^[6]^ The specific mechanism is unknown, but may be related to the location of the tumor. It has been documented that colon cancer can perforate the bowel and extend into the spleen, facilitating the direct spread of bacteria and subsequent abscess formation. The most frequently isolated bacteria in splenic abscesses include Escherichia coli, Streptococcus group D, Proteus mirabilis, Klebsiella pneumoniae and Bacteroides fragilis.^[7]^ In the present case, the bacterium identified was Bacteroides fragilis. Another explanation is that chemotherapy might worsen the immunosuppressive condition of cancer patients, erode mucosal barriers, and impair the capacity of the spleen to phagocytose cells.^[3]^ In this case, the patient presented with splenic metastasis, a rare occurrence. A study encompassing 1898 patients with solid malignant tumors identified splenic metastasis in only 57 (3%) cases. For patients with colon cancer, the reported incidence of splenic metastasis is approximately 2%.^[8]^ Another review of 6137 patients with metastases found splenic involvement in only 59 (0.96%) cases.^[9]^ The presence of splenic metastasis suggests an abundant blood supply and compromised immune function within the spleen, increasing the risk of bacterial invasion. In addition to the above characteristics, the patient with a splenic abscess in this report also had the following high-risk factors: immunocompromise resulting from long-term antitumor treatment, chronic hepatitis B infection with splenomegaly, and colonic mucinous adenocarcinoma with spleen metastasis.

Patients with splenic abscesses often present atypical clinical symptoms that are difficult to diagnose. The clinical signs included fever and chills, left upper quadrant abdominal pain, tender masses, nausea, and vomiting.^[10]^ The patient described herein had chills, fever, and slightly dull abdominal pain. Splenic abscesses are generally diagnosed by using imaging techniques. CT appears to be a sufficient technique to confirm splenic abscess if there is a strong clinical indication.^[11]^ However, some studies have shown that the sensitivities of CT and ultrasound are equally high.^[12,13]^ Once splenic abscesses are identified, splenectomy combined with antibiotic therapy is the most effective treatment for splenic abscesses^[14,15]^ especially in cases with partitioned, multiple, or complicated abscesses.^[16]^ However, in our case, anti-infection therapy was ineffective and the patient was in an extremely high platelet-consuming state. Moreover, splenectomy was not performed because severe thrombocytopenia is associated with a high risk of bleeding. Ultrasound-guided percutaneous splenic abscess perforation and drainage are less-invasive options. Nonetheless, this technique is suitable for treating isolated and thoroughly dissolved abscesses. After multidisciplinary discussion, we finally performed ultrasound-guided percutaneous puncture drainage. Eventually, the patient made good recovery.

Although spleen abscesses have been reported in patients with colon cancer, and splenic metastasis in colon cancer patients has also been documented, the concurrent occurrence of spleen abscess in a patient with colon cancer and splenic metastasis has not been previously reported. Additionally, the patient in this case exhibited hypersplenism due to hepatitis B, resulting in thrombocytopenia, which further complicates the treatment. The success of our case provides valuable clinical insights. Specifically, spleen metastasis in tumor patients necessitates vigilance for the potential development of spleen abscess; tumor patients with hepatitis B-induced hyperplenism require prolonged anti-tumor therapy, and spleen resection or splenic embolization may be considered at an appropriate juncture; a less invasive procedure can be feasible in cases of low platelet count without significant coagulation dysfunction; when alternative treatment options are limited, it is prudent to weigh the risks following multidisciplinary comprehensive evaluation and thorough patient communication. However, this is just 1 case; more cases are required to support these.

## Author contributions

**Investigation:** Zhangjun Qian, Rongfeng Zhu.

**Methodology:** Rongfeng Zhu, Yunlei Zhang.

**Writing – original draft:** Fang Cui.

**Writing – review & editing:** Yunlei Zhang.
